# Supplementary material for: The Effect of PEGDE Concentration and Temperature on Physicochemical and Biological Properties of Chitosan
Source: Polymers (Basel). 2019 Nov 7;11(11):1830. doi: 10.3390/polym11111830 (PMC6918179; doi:10.3390/polym11111830)
Supplement: Supplementary file 1 [file polymers-11-01830-s001.pdf]

The Effect of PEGDE Concentration and Temperature on Physicochemical and Biological Properties of Chitosan

Martha Gabriela Chuc-Gamboa[1], Rossana Faride Vargas-Coronado[1], José Manuel Cervantes-Uc[1], Juan Valerio Cauich-Rodríguez[1*], Diana María Escobar-García[2], Amaury Pozos-Guillén[2], Julio San Román del Barrio[3]

1 Unidad de Materiales, Centro de Investigación Científica de Yucatán, México. Calle 43 No. 130 x 32 y 34, Colonia Chuburná de Hidalgo, C.P. 97205, Mérida, Yucatán, México.

2 Laboratorio de Ciencias Básicas, Facultad de Estomatología, Universidad Autónoma de San Luis Potosí, México. Ave. Dr. Manuel Nava No. 2, Zona Universitaria, C.P. 78290 San Luis, S.L.P., México.

3 Instituto de Ciencia y Tecnología de Polímeros. España. Calle Juan de la Cierva, 3, C.P 28006 Madrid, España.

SUPPLEMENTARY DATA

S1. Suggested chemical reactions between CHT and PEGDE and CHT-GA

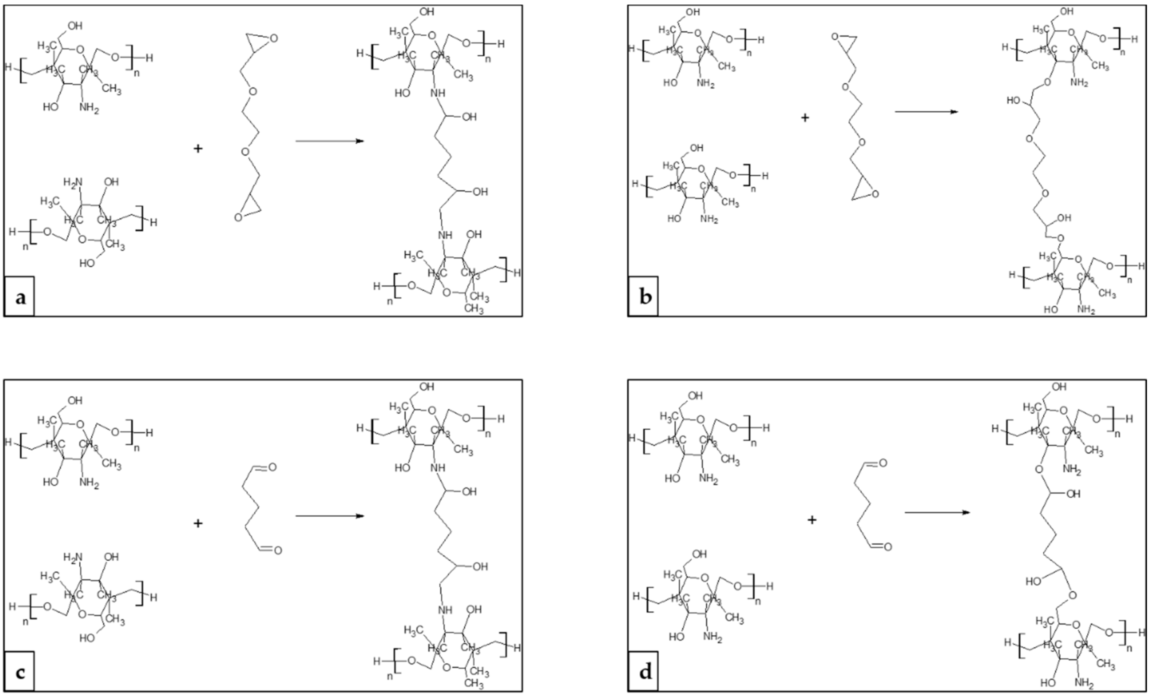

a) Expected crosslinking reaction between the NH_2 of chitosan and PEGDE. **b)** Expected crosslinking reaction between the OH of chitosan and PEGDE. **c)** Expected crosslinking reaction between the NH_2 of chitosan and GA. **d)** Expected crosslinking reaction between the OH of chitosan and GA.

S2. XPS scans for C, O and N

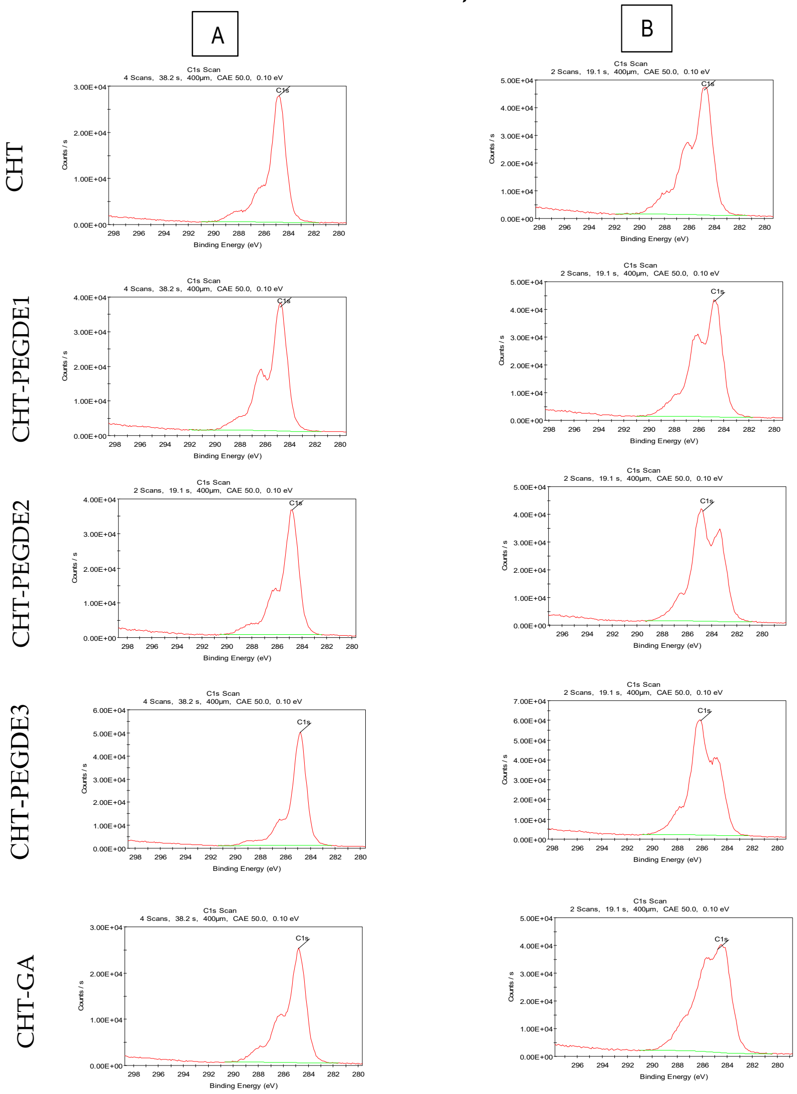

XPS spectrum of carbon on chitosan film dried at (A) 25°C (B) 150°C

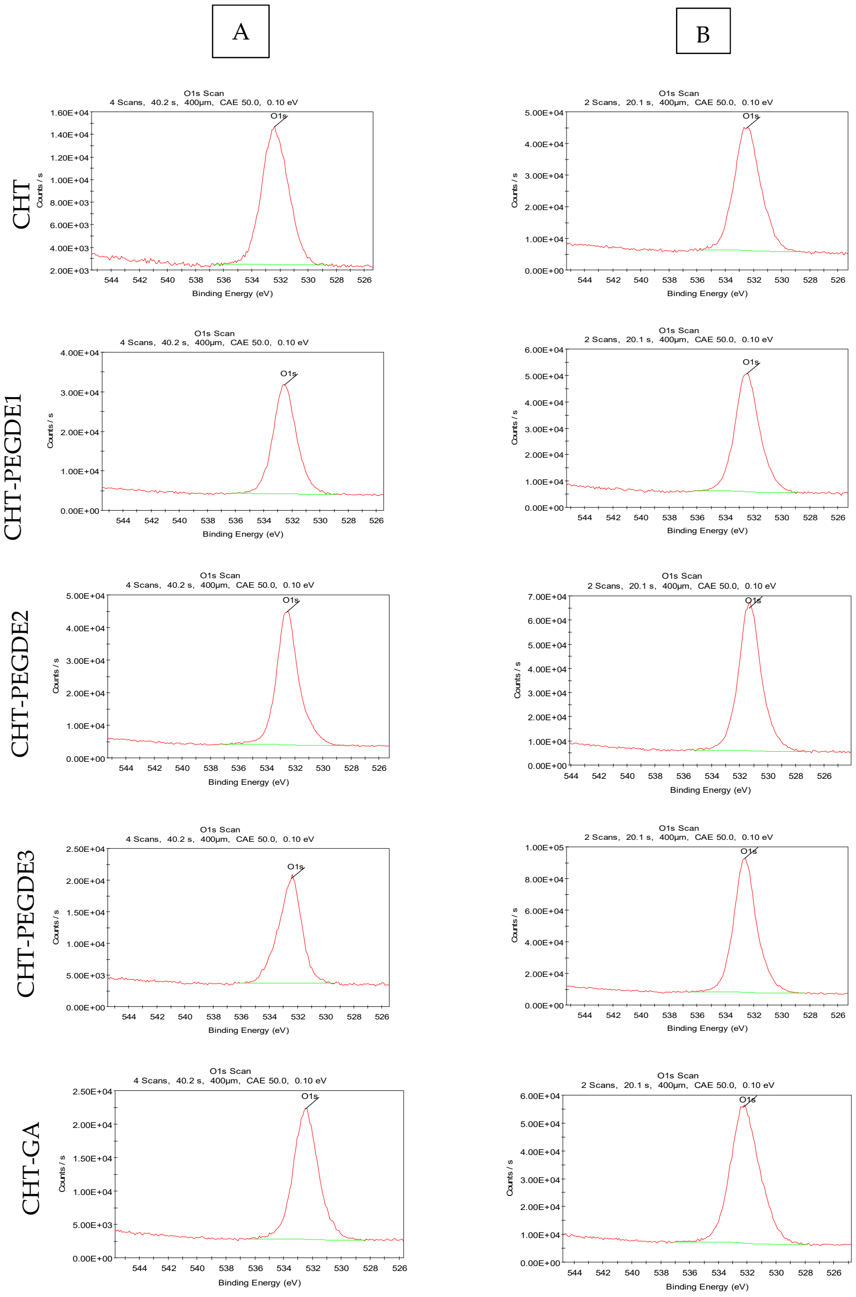

XPS spectrum of oxygen on chitosan film dried at (A) 25°C (B) 150°C

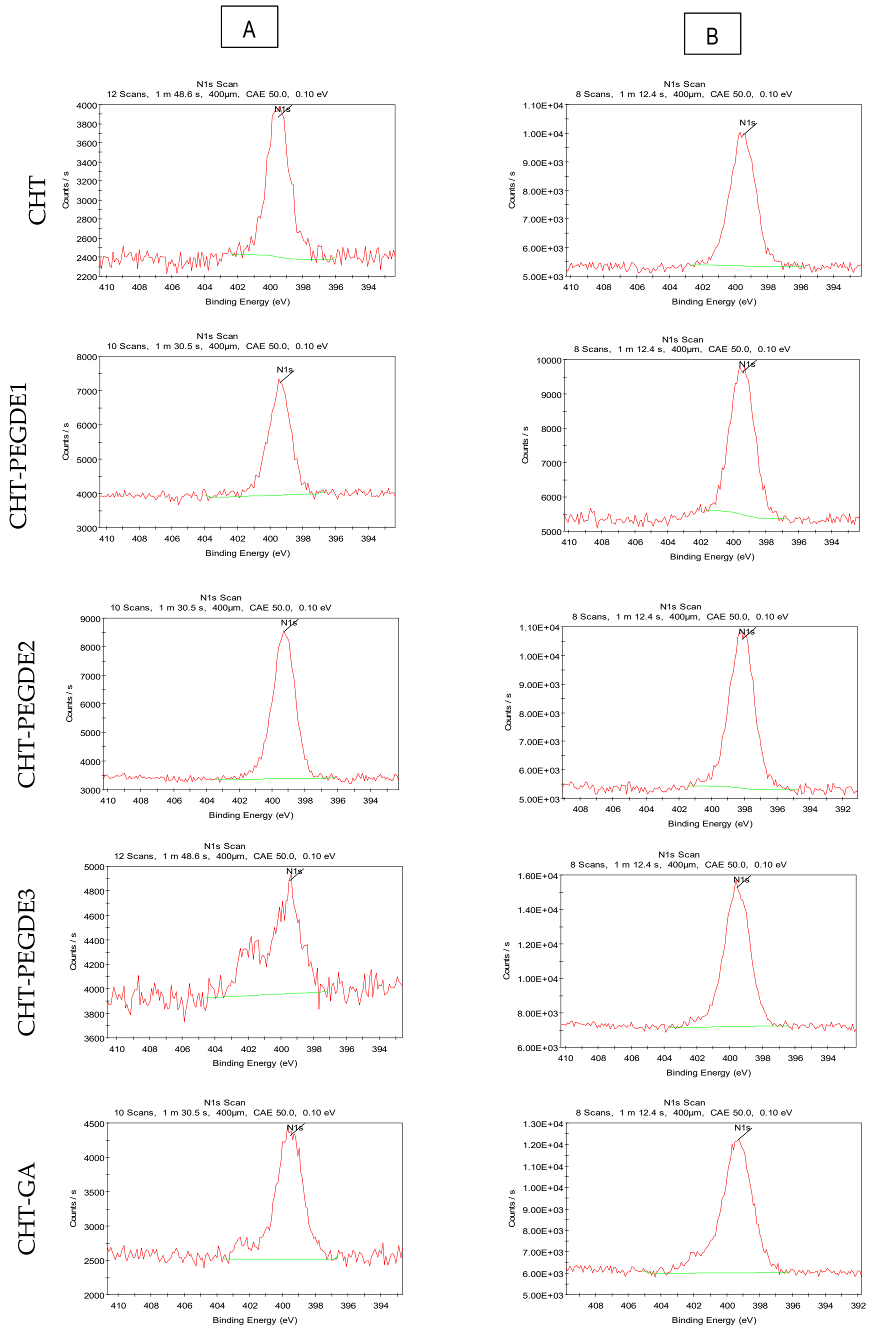

XPS spectrum of nitrogen on chitosan film dried at (A) 25°C (B) 150°C

S3 AFM 3D topography and roughness

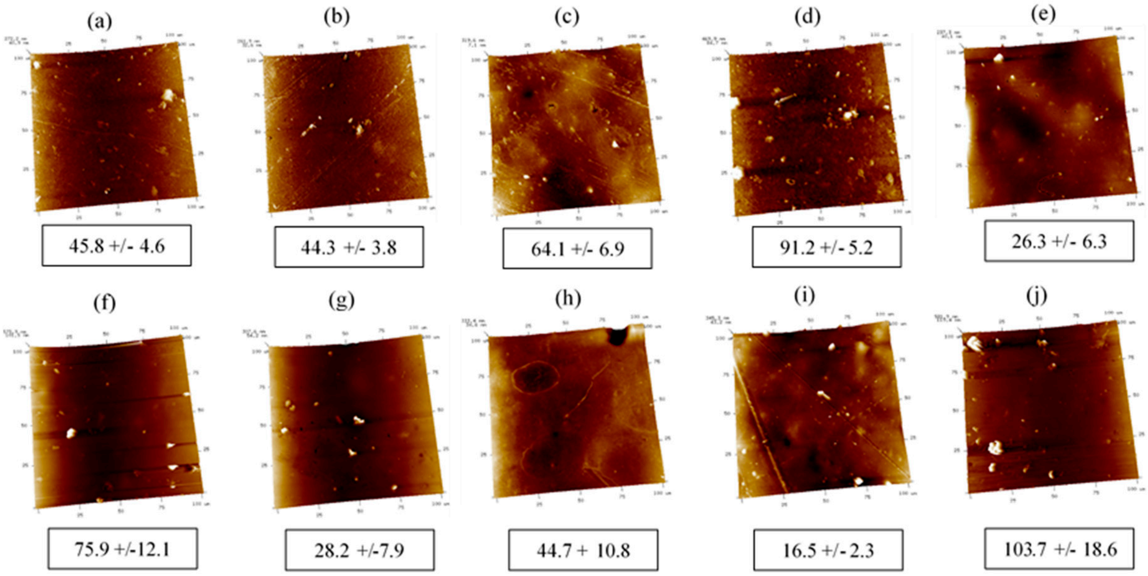

2D topography and Ra (nm) of the films dried at 25°C (Top, a - e) and additional dried at 150°C (bottom, f – j); a: CHT, b: CHT/PEDGE1, c: CHT/PEDGE2, d: CHT/PEDGE3, e: CHT/GA, f: CHT, g: CHT/PEDGE1, h: CHT/PEDGE2, i: CHT/PEDGE3, j: CHT/GA)
